# Evaluation of five different suture materials in the skin of the earthworm (*Lumbricus terrestris*)

**DOI:** 10.1186/2193-1801-3-423

**Published:** 2014-08-11

**Authors:** Melissa A Salgado, Gregory A Lewbart, Larry S Christian, Emily H Griffith, Jerry McHugh Law

**Affiliations:** Omaha’s Henry Doorly Zoo and Aquarium, Nebraska, 68107 USA; North Carolina State University College of Veterinary Medicine, North Carolina, 27607 USA; North Carolina Museum of Natural Sciences, Raleigh, North Carolina 27601 USA; Department of Statistics, North Carolina State University, North Carolina, 27695 USA

**Keywords:** Earthworm, Healing, Immune response, *Lumbricus terrestris*, Suture material

## Abstract

The purpose of this study was to determine which suture material is the most appropriate for dermal closure of terrestrial annelids. This paper describes the tissue reactions of the earthworm, *Lumbricus terrestris*, to five different types of suture materials in order to determine which suture material is the most appropriate for dermal closure. Silk, monofilament nylon, polydiaxonone, polyglactin 910, and chromic gut were studied. There was mild to moderate tissue reaction to all five suture materials. In all of the biopsies wound-healing reaction consisted of aggregates of blastemal cells which appeared in various stages of dedifferentiation from the body wall. Inflammatory cells infiltrated the wound sites, reminiscent of the typical foreign body reaction in vertebrates. The results indicate polyglactin 910 would be the best suture material with regards to tissue security and reaction scores. Chromic gut occupies the next position but there were problems with suture security over time. This appears to be the first suture material performance study on a terrestrial invertebrate. The earthworm, *Lumbricus terrestris,* was chosen for its wide availability, size, and the extensive species knowledge base. The earthworm may prove to be a good surgical/suture model for economically important invertebrates such as mollusks, tunicates, and insect larval stages.

## Introduction

Invertebrate medicine is a relatively new field with little known regarding the medical and surgical aspects of this paraphyletic group. As more veterinarians are confronted with invertebrate clinical and research challenges in laboratory and zoological settings, it will be increasingly important to develop sound surgical principles for these animals. This paper investigates the tissue reactions of a terrestrial invertebrate, *Lumbricus terrestris*, to five different types of suture materials as one step toward refining surgical techniques in invertebrates.

The purpose of this study was to determine which suture material is the most appropriate for dermal closure of terrestrial annelids. The earthworm was chosen for its wide availability, size, and our knowledge base regarding this species (Lewbart 2012). In previous studies regarding transplantation immunity in earthworms, it was demonstrated that earthworms readily accept integument autografts but always reject xenografts (Bailey et al. 1971; Cooper 1968; Cooper 1969a; Cooper 1969b; Cooper & Roch 1986). Therefore, earthworms have a cell-mediated immune system, and are also capable of adaptive immunity (i.e., specificity and memory).

The following suture materials were investigated: 4–0 silk, monofilament nylon, polydiaxonone, polyglactin 910, and chromic gut. An ideal suture material would provide good knot security, resist bacterial growth and infection, and react minimally with tissue (Fossum 2007). Tissues respond to sutures as they would any foreign material. If the inflammatory reaction to a specific suture material is reasonable, then the friability of the suture material increases, which in turn causes the sutures to fall out prematurely and increases the risk of infection and dehiscence (Stashak & Yturraspe 1978). To our knowledge, this is the first suture viability/efficacy study performed on a terrestrial invertebrate. Studies on the tissue reaction of different suture materials have been performed in cats, dogs, birds, koi fish, sea turtles, snakes, frogs, rats, and the sea hare (Anderson et al. 2010; Bellenger 1982; Bennett et al. 1997; DeNardo et al. 1996; Freeman et al. 1987; Govett et al. 2004; Greenwald et al. 1994; Hurty et al. 2002; Mcfadden et al. 2013; Park et al. 2013; Postlethwait 1970; Tuttle et al. 2002; Varma et al. 1981; Wood et al. 1984). Some of these studies indicate that suture materials are tolerated similarly among species. However, there are marked anatomical and environmental differences in annelids that may result in distinctive findings. These differences include the fact that an earthworm’s immune system and wound healing reaction are unlike that of vertebrate species in which suture studies have been performed. This variation may affect the severity of tissue reaction and absorption of different suture materials. Earthworms move primarily by muscular peristaltic contractions; therefore, suture material may not hold well. Earthworms also move freely through a variety of substrates, including dirt, mud, sand, decaying leaves, and even water. A clean environment may not be achieved after surgery and earthworms could be exposed to many potential pathogens that may invade the surgical site and compromised dermis.

## Materials and methods

### Ethics statement

This study was not under the prevue of an IACUC. Handling and sampling procedures were consistent with standard vertebrate protocols and veterinary practices.

### Animals

Seventy-two earthworms, *Lumbricus terrestris*, were obtained from a commercial vendor for this study. They were randomly assigned to groups of 12, each housed in a dark plastic container with a substrate of damp soil and dead leaves. The enclosure was kept at a temperature of 20°Celsius and at 30% soil moisture content, based upon published accounts (Berry & Jordan 2001; Braun et al. 2006; Cooper 2004). They were allowed to acclimate for 1 week prior to initiating the study.

### Anesthesia protocol

Each earthworm was anesthetized by immersion in 5% ethanol solution for 5 minutes (Cooper 1968; Cooper & Roch 1986; Stevenson & Beane 2010). The earthworms were then rinsed off in anesthetic-free, dechlorinated tap water at room temperature. The incision site was prepared by gently wiping the area with a sterile cotton swab.

### Suture application

Surgery was performed using aseptic technique. A #15 scalpel blade was used to make a stab incision along the animal’s caudal third of the body, full thickness, through the skin. Five of the six groups were assigned to have one of the following sutures (all from the same manufacturer) placed: 4–0 silk, monofilament nylon (Ethilon), polydioxanone (PDS II), polyglactin 910 (Vicryl), chromic gut (Ethicon, A Johnson & Johnson Company, Somerville New Jersey 08876 USA). A single, simple interrupted suture was placed at the site of the stab incision. One group was assigned as a control, in which a single stab incision was performed, but no suture material was placed and healing was by second intention. The earthworms recovered from anesthesia by placing them in a shallow pool of clean water before return to their respective enclosures. The surgical sites were examined daily for gross signs of dehiscence and inflammation and graded on a scale of 0 to 5 (Table 1).

**Table 1:** **System for scoring gross and histologic tissue reaction to suture**

Gross scoring system	
Score	Description
0	No deviation from incised but non-sutured site
1	Minimal edema, inflammation, and epidermal changes
2	Mild edema, inflammation, and epidermal changes
3	Moderate edema and inflammation, +/- scattered areas of necrosis
4	Moderately severe edema and inflammation, with many areas of necrosis
5	Severe edema, inflammation, and epidermal changes
**Histologic scoring system**	
**Score**	**Description**
0	No remarkable microscopic changes
1	Minimal changes, including inflammation and organization of wound reaction
2	Mild inflammation; well-organized wound reaction of blastemal cells
3	Moderate inflammation; more fulminant blastemal cell reaction
4	Moderately severe microscopic changes (as described above)
5	Severe microscopic changes (as described above)

### Biopsy procedures and processing

Three days after surgery, six earthworms were randomly selected from each of the six groups and anesthetized as previously described. A single 3 mm full-thickness punch biopsy of the skin at the incision site was taken, with the sutures in situ. This procedure was repeated 6 days after surgery with the remaining six earthworms from each group. The biopsy sites were left to heal by second intention with no further suture placement.

The tissue samples collected were immediately fixed in 10% neutral buffered formalin, processed routinely, embedded in paraffin, sectioned at 5 μm, and stained with haematoxylin and eosin (H&E) for examination by light microscopy. The tissue samples were randomized and evaluated blindly by a single pathologist, who scored each of the biopsies on a scale of 0 to 5 based on degree of inflammation, epidermal changes, and edema (Table 1). The score for the control was subtracted from each suture material score to obtain the overall reaction score.

### Statistical methods

The histological grades (0–5) were analyzed using ordinal logistic regression in SAS (Version 9.3, Cary, NC). The odds of a lower histological score were modeled as a function of both time (day 3 and day 6) and suture material. Exact 95% confidence intervals were calculated and compared for the percentage of sutures retained in each group at both days 3 and 6.

## Results

### Suture study

There was not a statistically significant time effect in the model (p = 0.228), but there were statistically significant differences between suture materials (p = 0.0344). Chromic gut, Ethilon, and Vicryl all have higher probabilities of lower histological scores than the control group (p = 0.003, 0.008, and 0.006, respectively), but do not differ significantly among themselves. PDS and silk sutures did not differ significantly from the control group (p = 0.064 and p = 0.160, respectively). There were no statistically significant differences in the percentages of sutures retained per group, but that can be attributed to small sample sizes within each group. Chromic gut, Ethilon, and PDS all have statistically significantly less than 100-percent suture retention at day 6.

**Figure 1:**
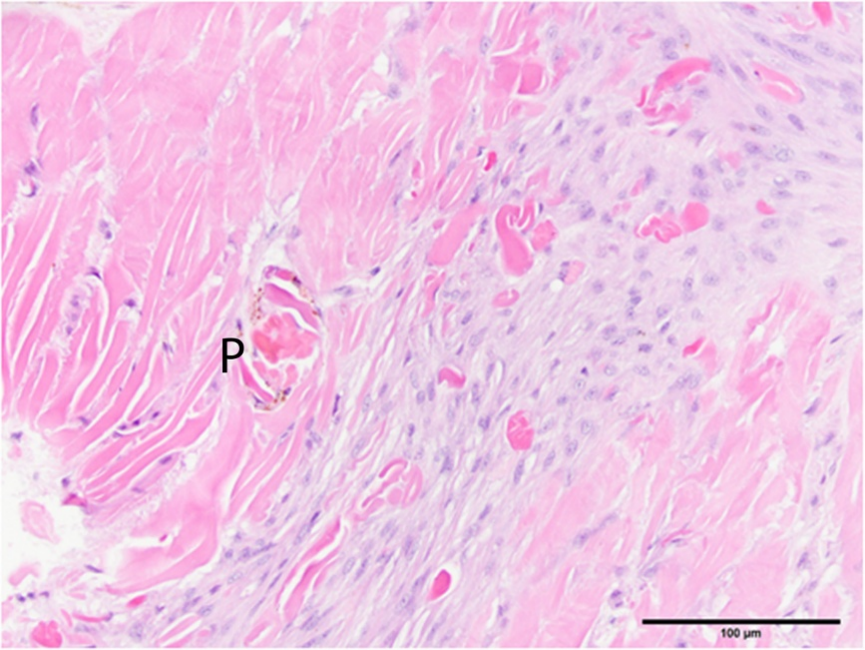
**Grade 2 reaction to 4–0 polyglactin 910 suture in the earthworm (** ***Lumbricus terrestris*** **) body wall, showing a relatively mild blastema cell reaction with no visible infiltration of inflammatory cells.** A small amount of golden brown pigment (P) at one edge indicates wound maturation. Hematoxylin and eosin stain.

As evident by the results in Tables 2 and 3, there was mild to moderate gross or microscopic response to all five suture materials. The greatest variability occurred within the gross results with scores ranging from 0.5 (silk Day 3 and Day 6) to 3.5 (polydioxanone Day 6). Polydioxanone also had a high Day 3 gross score of 2.5. Furthermore, in the polydioxanone groups, 2/6 individuals lost sutures at Day 3 and 5/6 lost sutures at Day 6. Silk displayed the most consistent results with identical gross and microscopic scores for the Day 3 and 6 biopsies (no lost sutures). Polydioxanone and polyglactin 910 displayed consistent Day 3 and 6 histopathology scores of 3.0 and 2.5, respectively. It should be noted that 3/6 individuals in the Day 6 nylon group lost sutures. Polyglactin 910 showed low gross scores of 1.0 and 0 (Day 3 and 6 respectively) and all sutures were retained. The chromic gut sutures produced low gross scores (1.5) with a moderate increase in the microscopic scoring over time (2.50 to 2.83). Two individuals in the chromic gut Day 6 group lost sutures. As shown in Table 3, polyglactin 910 (Vicryl) had the lowest mean histologic severity grades, 2.50 at both time points (Figure 1), which were very close to the histologic grades of the controls. Chromic gut was the next least reactive suture material type, with mean histologic grades of 2.50 at 3 days and 2.83 at 6 days.

**Table 2:** **Median overall gross suture reaction scores associated with five different suture materials in the body wall of earthworms 3 and 6 days after surgery**

Suture type	3 days	6 days
	Gross	Gross
**Silk**	0.5	0.5
Monofilament nylon*	1.5	3
Polydioxanone**	2.5	3.5
**Polyglactin 910**	1	0
Chromic gut***	1.5	1.5

*Three individuals in the nylon group lost sutures by Day 6.

**Two individuals in the polydioxanone group lost sutures by Day 3 and five individuals lost sutures by Day 6.

***Two individuals in the chromic gut group lost sutures by Day 6.

**Table 3:** **Individual and mean histologic grade reaction scores associated with five different suture materials in the body wall of earthworms 3 and 6 days after surgery**

Suture reaction histologic grades (range = 0–5)								
	*Sample #:	#1	#2	#3	#4	#5	#6	
	**Slide #							Mean severity grade:
Silk	1A	3	2	3	3	3	2	2.67
	1B	4	4	4	2	3	4	3.50
Polydioxanone	2A	2	3	4	4	3	2	3.00
	2B	2	3	4	4	3	2	3.00
Polyglactin 910	3A	3	3	2	2	3	2	2.50
	3B	2	2	2	2	4	3	2.50
Chromic gut	4A	2	2	3	4	2	2	2.50
	4B	2	3	3	4	3	2	2.83
Nylon	5A	2	3	3	4	2	2	2.67
	5B	4	3	3	3	4	2	3.17
Control	6A	2	2	3	2	3	2	2.33
	6B	3	2	2	3	3	2	2.50

*Each slide (1A, 1B, etc.) contained six biopsies = one from each animal.

**Group A (1A, 2A, etc.) was sampled 3 days after surgery; group B, 6 days after.

**Figure 2:**
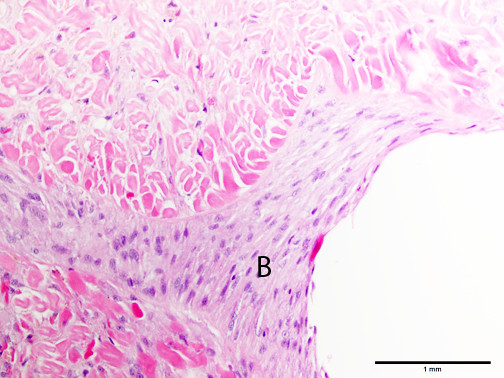
**Cellular reaction in the body wall of the earthworm,** ***Lumbricus terrestris*** **, to 4–0 silk suture, showing layers of basophilic blastema cells (B) with round to oval to elongated nuclei and indistinct cytoplasmic borders.** Hematoxylin and eosin stain.

**Figure 3:**
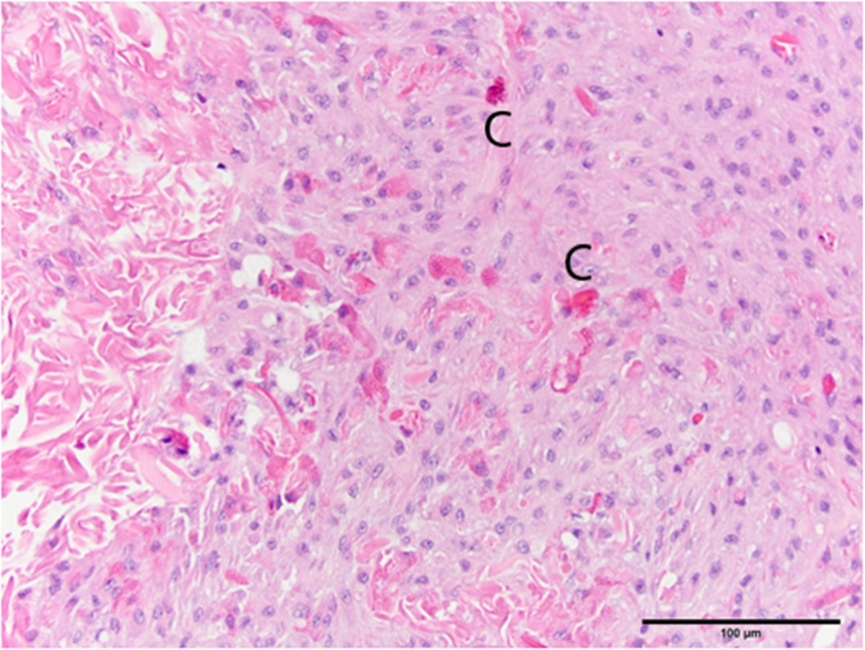
**Grade 4 reaction to 4–0 polydioxanone suture in the earthworm (** ***Lumbricus terrestris*** **) body wall, showing infiltration of numerous eosinophilic granulocytes (coelomocytes, C) among a background of pale basophilic blastema cells.** Hematoxylin and eosin stain.

**Figure 4:**
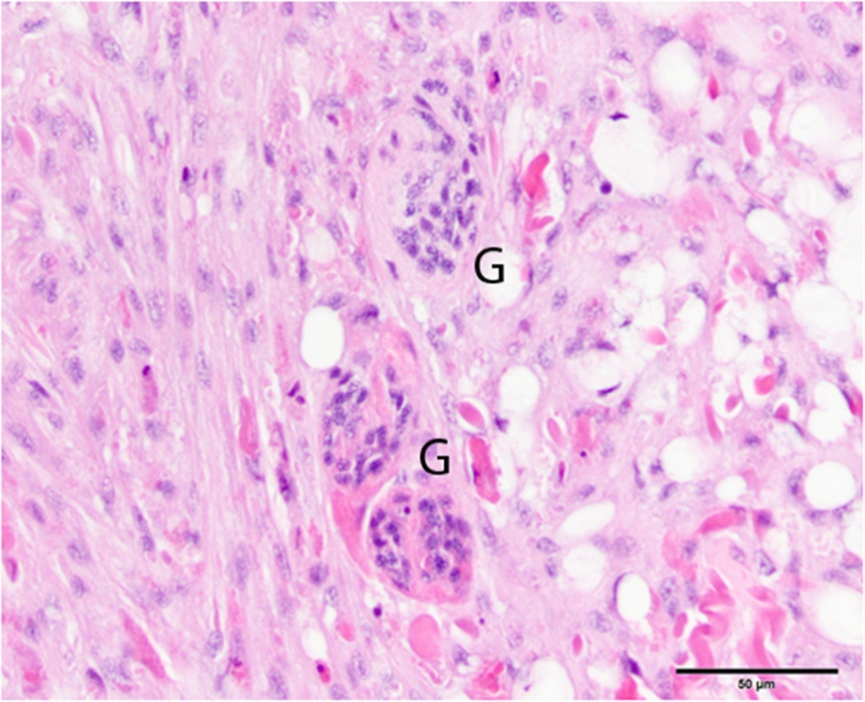
**Grade 3 reaction to 4–0 nylon suture in the earthworm (** ***Lumbricus terrestris*** **) body wall, showing three multinucleate giant cells (G) at the edge of the blastema cell reaction to the left.** Hematoxylin and eosin stain.

The microscopic changes in reaction to wounding consisted of aggregates of pale, basophilic blastemal cells and variable amounts of extracellular matrix that provided the wound closure, along with variable infiltrates of inflammatory cells. Interestingly, edema did not appear to be a prominent feature of the wound reaction (less of a vascular reaction than mammals). The blastemal cells had relatively large, round to oval nuclei and indistinct cell borders (Figure 2), whereas the inflammatory cells were predominantly coelomocytes with brightly eosinophilic cytoplasmic granules (Figure 3); these latter cells appear similar to mammalian and piscine eosinophils on an H&E stain, but were called acidophils by Stein et al. using Wright’s stain (Stein et al. 1977). A few tissue sections had small numbers of multinucleated giant cells, similar to suture reactions in vertebrates (Figure 4).

All 72 earthworms survived the study.

## Discussion

In all of the earthworm biopsies in our study, the wound healing reaction consisted of aggregates of blastemal cells that appeared in various stages of transition (dedifferentiation) from the skeletal muscle of the body wall. This is consistent with a recent paper by Park et al. who demonstrated using another annelid (*Eisenia andrei*) that the major origin of blastemal cells is the longitudinal muscle layer of the body wall (Park et al. 2013). Variable numbers of inflammatory cells (coelomocytes), including scattered multinucleate giant cells, infiltrated the wound sites, reminiscent of the typical foreign body reaction in vertebrate animals. However, the tissues lacked the neovascularization and accompanying edema that characterizes wound healing in vertebrates. With time, the blastemal cells will redifferentiate into the various mature cell/tissue types, including epidermis (Park et al. 2013).

Our results suggest that polyglactin 910 may be the best choice for terrestrial annelids. Its low gross scores and relatively mild/bland microscopic changes as compared to controls may indicate it’s a good choice for other invertebrates with similar life styles. In addition all sutures were retained. This and monofilament suture materials produce the least amount of tissue reaction in several avian and domestic mammalian species (Bennett et al. 1997; DeNardo et al. 1996; Knowles 1976; Wood et al. 1984). Similar results were reported in fish, amphibians, and reptiles. Polyglyconate, a synthetic, absorbable, monofilament material, produced the least tissue reaction in koi carp (*Cyprinus carpio*), when compared with four other suture materials (Hurty et al. 2002). Consistent with these findings were favorable tissue reactions from monofilament nylon in African clawed frogs (*Xenopus laevis*) (Tuttle et al. 2002) and poliglecaprone (Sanz et al. 1988) and polyglyconate in juvenile loggerhead turtles (*Caretta caretta*) (Govett et al. 2004). Polyglyconate performed well to close a skin excisional biopsy site in a cuttlefish (*Sepia officinalis*); the sutures dislodged/dissolved on their own (Harms et al. 2006). Interestingly, this was not the case in another invertebrate, *Aplysia californica*, where silk, a braided organic material, was superior to the monofilament sutures examined (Anderson et al. 2010).

In the current study, gross silk scores were acceptable, consistent, and the sutures remained in place, but the mean histologic grade increased to 3.5/5.0 at the longer time point. These findings are in parallel with the aforementioned *A. californica* study with regards to the gross findings. While not a suture material efficacy study per se, a report on anchoring the medicinal leech (*Hirudo medicinalis*) with superficial and deep tissue braided silk found no difference in survival among these two groups and a non-sutured control group (Davila et al. 2009).

The chromic gut gross scores at Days 3 and 6 were identical (1.5) and indicate only moderate inflammation. Two subjects lost suture by Day 6, a possible disadvantage, and a reason to rank silk as a slightly better option.

The nylon gross and microscopic tissue reaction scores were mild to moderate at all time points. The Day 6 gross score of 3.0 was second in severity only to polydioxanone, which had the disadvantage of significant suture loss at both biopsy time points (Table 2). Based on these performance scores nylon and polydioxanone would be ranked in the lower tier of the five suture materials examined.

The findings of this study indicate polyglactin 910 would be the best suture material with regards to tissue security and reaction scores. Chromic gut occupies the next position due to poorer suture security over time.
